# SNP-guided identification of monoallelic DNA-methylation events from enrichment-based sequencing data

**DOI:** 10.1093/nar/gku847

**Published:** 2014-09-18

**Authors:** Sandra Steyaert, Wim Van Criekinge, Ayla De Paepe, Simon Denil, Klaas Mensaert, Katrien Vandepitte, Wim Vanden Berghe, Geert Trooskens, Tim De Meyer

**Affiliations:** 1 Department of Mathematical Modelling, Statistics and Bioinformatics, University of Ghent, Ghent 9000, Belgium; 2 Department of Biology, University of Leuven, Leuven 3000, Belgium; 3 PPES, Department of Biomedical Sciences, University of Antwerp, Wilrijk 2610, Belgium

## Abstract

Monoallelic gene expression is typically initiated early in the development of an organism. Dysregulation of monoallelic gene expression has already been linked to several non-Mendelian inherited genetic disorders. In humans, DNA-methylation is deemed to be an important regulator of monoallelic gene expression, but only few examples are known. One important reason is that current, cost-affordable truly genome-wide methods to assess DNA-methylation are based on sequencing post-enrichment. Here, we present a new methodology based on classical population genetic theory, i.e. the Hardy–Weinberg theorem, that combines methylomic data from MethylCap-seq with associated SNP profiles to identify monoallelically methylated loci. Applied on 334 MethylCap-seq samples of very diverse origin, this resulted in the identification of 80 genomic regions featured by monoallelic DNA-methylation. Of these 80 loci, 49 are located in genic regions of which 25 have already been linked to imprinting. Further analysis revealed statistically significant enrichment of these loci in promoter regions, further establishing the relevance and usefulness of the method. Additional validation was done using both 14 whole-genome bisulfite sequencing data sets and 16 mRNA-seq data sets. Importantly, the developed approach can be easily applied to other enrichment-based sequencing technologies, like the ChIP-seq-based identification of monoallelic histone modifications.

## INTRODUCTION

For diploid organisms, gene expression is denoted as monoallelic if only one allele is transcriptionally active. The expressed allele can be randomly selected (e.g. X-chromosome inactivation and some autosomal genes) or predetermined by parental imprinting (1–3). Erroneous monoallelic expression has been associated to several genetic disorders, like the Prader–Willi syndrome, as well as to certain forms of cancer, like Wilms’ tumour. Both diseases are caused by loss of imprinting of some genes in the 15q11-q13 and 11p15.5 region, respectively (4).

Epigenetics is defined as the study of inheritable modifications on both chromatin and DNA that have an influence on gene expression without changing the underlying DNA sequence (5). Mammalian DNA-methylation is an epigenetic mark that is predominantly found in a CpG sequence context (6). This methylation mark has been linked with gene expression and when located in the promoter region, it generally leads to transcriptional silencing of the corresponding gene (7). As it is a defining feature of cellular identity and essential for normal development, its dysregulation is often associated with disease (4). Monoallelic DNA-methylation is likely to bare an important role in the regulation of monoallelic expression (8). In addition to DNA-methylation, histone modifications also contribute to the maintenance of monoallelic expression. The methylated, silenced allele is mostly sustained with the repressive histone modification histone H3 trimethylation at lysine 9 (H3K9me3) while the active allele is characterized by the permissive histone marker H3 trimethylation at lysine 4 (H3K4me3) (9).

An important example of monoallelic DNA-methylation is the regulation of the parental-dependent monoallelic expression at imprinted loci, where the silenced allele is significantly more methylated than the active, expressed allele (2). Although imprinting is a well-investigated topic and several studies already provided evidence (e.g. computational predictions based on DNA sequence characteristics or detection of monoallelic expression) of some regions with monoallelic DNA-methylation (2,3,10–16), only a few imprinted regions are well characterized in humans, like, for example, the *IGF2/H19* region. Furthermore, monoallelic methylation has recently been recognized as very common at non-imprinted loci affecting autosomal genes, regulating, for example, the production of specific antibodies and receptors in the immune system as well as the selection of olfactory receptors (17,18). While monoallelic methylation has been shown to play an important role in the differentiation between tissues, little is known about the specific location of these loci as well as the genome-wide character of monoallelic DNA-methylation.

The recent advent of next-generation massively parallel sequencing platforms has introduced the possibility of genome-wide DNA-methylation profiling. Bisulfite sequencing, which combines bisulfite treatment of genomic DNA (gDNA) with the high-throughput sequencing of the entire genome, is the gold standard and allows to readily identify monoallelic methylated alleles (19), but is very costly and therefore outside the reach of smaller projects. Fortunately, cost-effective alternatives based on the specific enrichment of methylated portions of the genome (i.e. enrichment-based methods) such as methylated DNA immunoprecipitation followed by sequencing (MeDIP-seq) and methyl-CpG binding domain protein sequencing (MethylCap-seq) exist. Yet, these methods do neither provide single base pair (bp) resolution nor information regarding unmethylated alleles and are therefore not directly applicable to detect monoallelic events (20). While some approaches already tried to tackle this issue, they rely on the combination of multiple sequencing technologies, like, for example, the integrative method of Harris *et al.* (21), which tries to find regions with intermediate and potentially monoallelic events by combining data originating from MeDIP-seq, methylation-sensitive restriction enzyme sequencing (MRE-seq), ribonucleic acid sequencing (RNA-seq) and chromatin immunoprecipitation followed by sequencing (ChIP-seq).

To circumvent these issues, we developed a data analytical framework that solely uses data from enrichment-based sequencing (like MethylCap-seq), which screens for regions that exhibit monoallelic DNA-methylation based on classical population genetic theory, i.e. the Hardy–Weinberg equilibrium, in a parental-independent and genome-wide manner. This theory states that in a large random-mating population with no selection, mutation or migration both the allele and genotype frequencies of a gene locus with two alleles are constant from generation to generation, and furthermore, that there is a simple relationship between these allele and genotype frequencies: if the alleles are *A* and *a* with frequencies *p* and *q* (= 1 − *p*), respectively, then at equilibrium the genotype frequencies of *AA*, *Aa* and *aa* are *p^2^*, 2*pq* and *q^2^*, respectively (22).

The developed pipeline first compares enrichment-based sequencing data of multiple samples to the public NCBI Single Nucleotide Polymorphism (SNP)-archive (dbSNP) in order to screen the obtained non-duplicate, uniquely mappable sequence reads for SNPs. Only SNP loci with an adequately coverage and allele frequency are retained and the effect of sequencing errors is further reduced by comparing the chance of a sequencing error with the chance of detecting genuine SNPs. For each single SNP locus, the Hardy–Weinberg theorem is then applied to evaluate whether the observed frequency of samples featured by a biallelic event is lower than randomly expected (22). Using a permutation approach, confidence limits are simulated and genomic regions with a *P*-value smaller than the *P*-value corresponding with a given false discovery rate (FDR) can be assumed to harbour a monoallelic event.

Starting from MethylCap-seq data of a mixture of 334 Caucasian human samples and an FDR of 0.1, this methodology allowed the identification of 80 monoallelically methylated loci, significantly more found than expected in promoter regions. Of these 80 loci, 25 have previously been linked to imprinting. Additional validation was done using both 14 whole-genome bisulfite sequencing (WGBS) data sets of diverse origin and mRNA-seq data of 16 normal tissues. Here, the analysis was performed on available samples originating from a variety of tissues, mostly cancer tumours, providing a challenging data set to identify monoallelic methylation events (see Discussion). However, even in this set-up generally known imprinted regions were identified as well as putative novel imprinted genes, demonstrating the robustness of our method. Finally, because of the general rationale of the developed approach, it can be applied to enrichment-based sequencing applications to detect monoallelic features other than DNA-methylation. A possible application could be ChIP-seq (23) to screen for monoallelic histone modifications (24–28).

## MATERIALS AND METHODS

### Samples

A total of 334 human samples, mostly cancer samples of various tissues, was used to detect monoallelically methylated loci (Supplementary Table S1). Of these 334 samples, 215 samples were of female origin and only these were used to analyse the X-chromosome. gDNA was extracted from these samples with the Easy DNA kit (Invitrogen K1800-01) using protocol #4 from the manufacturer manual. The DNA concentration was measured on a Nanodrop ND-1000. Subsequently, the gDNA was sheared on the Covaris S2 with following settings: duty cycle 10%, intensity 5, 200 cycles per burst during 180 s to obtain fragments with an average length of 200 bp. The power mode was frequency sweeping, temperature 6°C–8°C and water level of 12. A total of 500 ng was loaded in 130 μl TE (1:5) in a microtube with Adaptive Focused Acoustics (AFA) intensifier.

### Methyl-CpG binding domain sequencing

Methyl-CpG binding domain protein sequencing (MethylCap-seq) (20), which combines enrichment of methylated DNA-fragments by methyl-CpG binding domain (MBD)-based affinity purification with massively parallel sequencing, was used to profile the DNA-methylation pattern of the 334 samples. The samples were sequenced according to the protocol described in the paper of De Meyer *et al.* (29) with some additional modifications: (i) After DNA fragmentation, the methylated fragments were captured using Diagenode's MethylCap kit starting from a DNA concentration of 500 ng instead of 200 ng. (ii) Paired-end sequencing was done on either the Illumina GAIIx or the HiSeq platform. Depending on the sequencing platform, the obtained paired-end sequence reads were 45 or 50 bp, respectively.

### Data pre-processing

**Figure 1.**
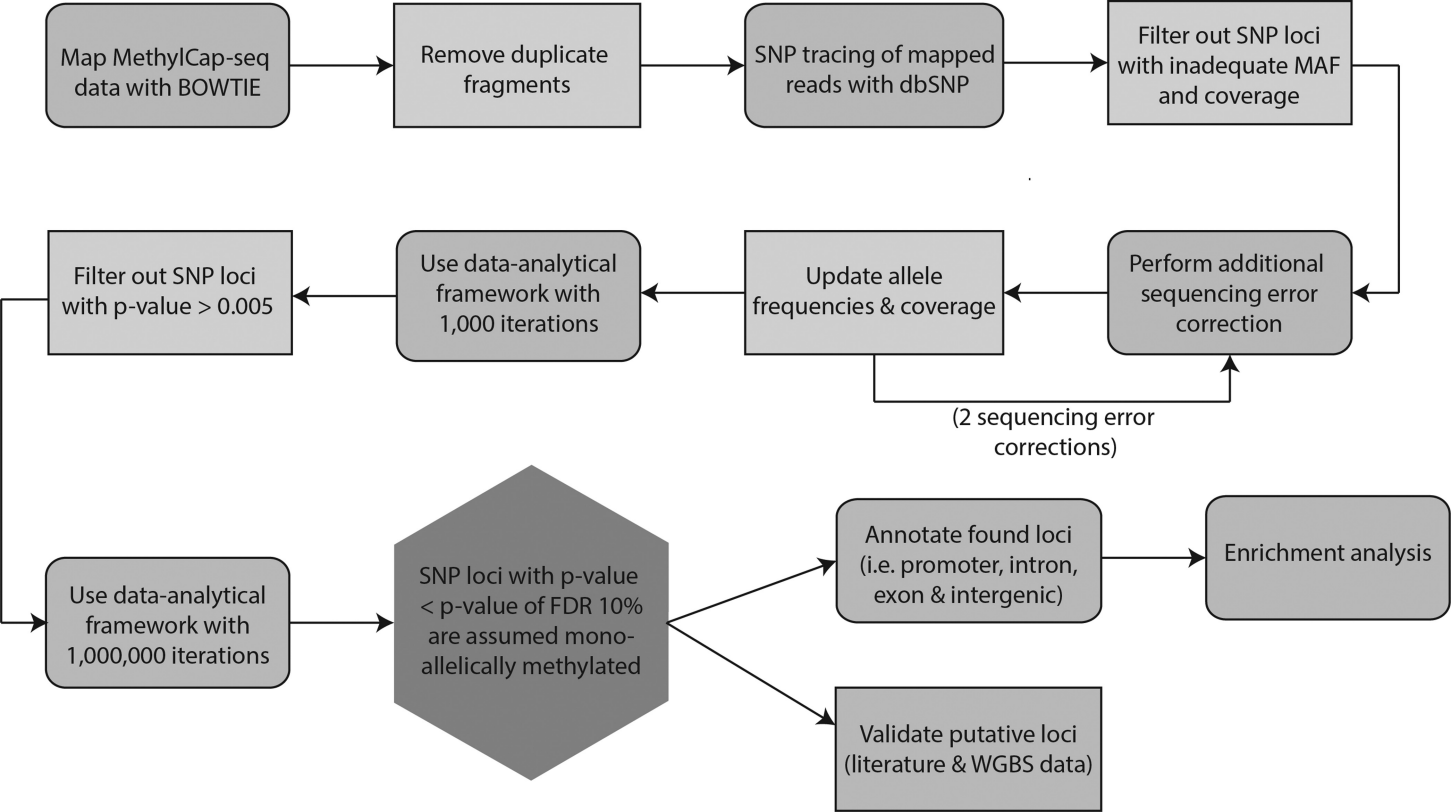
Overview of bioinformatics pipeline to detect putative monoallelically methylated SNP loci starting from MethylCap-seq data. After mapping with BOWTIE the non-duplicate, uniquely mapped reads are screened for SNPs using dbSNP. To reduce the computational load SNP loci with a too high MAF and/or a too low overall coverage are filtered. In this reduced data set, an additional sequencing error correction was performed with two iterations. The corrected data was next put in the newly developed data-analytical framework with 1000 and 1 000 000 iterations, respectively. Only loci that obtained a *P*-value smaller than or equal to 0.005 after the first iteration were kept as input for the second iteration. If the *P*-value obtained for a locus was smaller than the *P*-value corresponding with an FDR of 0.1 the monoallelic methylation on this locus was called significant. After determining the functional annotation of these SNP positions an enrichment analysis was performed. Finally, the resulting loci were validated using both literature and WGBS data.

The rationale behind the proposed methodology is that biallelic DNA-methylation results in MethylCap-seq data which is in Hardy–Weinberg equilibrium for each locus, i.e. if SNPs are present for a locus, both homozygous and heterozygous subjects will be detected at a predictable rate (22). However, in case of monoallelic methylation, heterozygous samples will no longer be detected resulting in deviation from the Hardy–Weinberg equilibrium, which can be measured. For a detailed description of the statistical framework, see the Additional Methods. Figure 1 gives an overall representation of the workflow starting from MethylCap-seq data.

#### Mapping

For each of the 334 samples, the MethylCap-seq paired-end reads were mapped using BOWTIE (v1.0.0) (30). The mapping parameters were chosen so that only those paired-end reads that mapped uniquely on the human hg19/GRCh37 reference assembly within a maximum of 400 bp of each other were retained. In order to both reduce the presence of sequencing errors as well as to allow the occurrence of real SNPs, a maximum of three mismatches was allowed. As suggested by Stevenson *et al.* (31), allowing three mismatches during the alignment step significantly reduces the reference allele bias at SNP loci while still enabling an ample amount of uniquely mapped reads. Duplicate fragments, i.e. fragments with the exact same location of both paired-end reads, were disposed as these are most likely the result of amplification of the same sequence reads during the library preparation.

#### SNP tracing

The non-duplicate, uniquely mappable reads were subsequently screened for SNPs. Only positions that showed a mismatch in the mapping of one or more samples and that overlapped known single nucleotide variations (SNVs) of the dbSNP (version 137) were withheld. Not keeping all the mismatches reduces both the effect of sequencing errors (false positives) and the computational load in the further analyses. Also, for each locus, the coverage of each SNP variant was determined, and the allele frequencies were estimated.

#### Additional data filtering and correction

Both for computational reasons and as a first filtering step for sequencing errors, SNP loci with a very high major allele frequency (MAF) were filtered (threshold 0.9). Additionally, a minimal total coverage threshold, i.e. across all samples, for each SNP locus was imposed (350 ∼ 1× per sample). Note that loci not fulfilling both criteria are unlikely to provide sufficient power for the subsequent statistical analysis. As analysis of the X-chromosome involved fewer samples, the threshold for the coverage was set to a less stringent value, namely, 250 instead of 350, which roughly corresponded to the number of female subjects.

In this reduced data set, an additional sequencing error correction was performed. For computational reasons, a simple Bayesian methodology was implemented. Basically, for each sample and locus, the chance of obtaining a certain profile was calculated under (i) the assumption of heterozygosity and (ii) the assumption of homozygosity but with additional sequencing errors. The option with the largest *a posteriori* change was withheld (with alleles representing putative sequencing errors being removed from the data set). As the prior chances of homozygosity and heterozygosity were based on the allele frequencies, which are updated upon each round of the Bayesian algorithm, this method was performed twice (see Additional Methods Section 2.1.3). This approach can be considered to be conservative (i.e. to disfavour the presence of monoallelic DNA-methylation), as (i) only two rounds of correction were applied and (ii) the sequencing error estimate (0.25%, based on Quail *et al.* (32)) is on the lower bound of estimates reported and is based on the performance of the Illumina HiSeq, whereas also more error prone GAIIx data were included in this study.

### Detection of monoallelically methylated loci

After additional filtering and data correction, the remaining data were used as input of the new data-analytical framework developed in the R statistical environment (R 2.15.2). The statistical strategy and practical implementation are elaborated in the Additional Methods. In summary, based on the observed allele frequencies, theoretically expected genotype frequencies can be calculated assuming Hardy–Weinberg equilibrium in the overall data set. If the observed frequency of heterozygote individuals is significantly reduced relative to Hardy–Weinberg expectations, this indicates significant monoallelic methylation. Null distributions were generated using random data with the same allele frequencies and sample coverages (for that locus) as in the original data. This approach accounts for the increased likelihood of erroneously calling loci with a low coverage homozygous. *P*-values were determined by comparison of the observed frequency of heterozygotes with the generated null distributions. Only loci that obtained a *P*-value smaller than or equal to 0.005 after the first iteration were kept as input for the second iteration. Thus, after the first iteration round, loci that were in all probability not monoallelically methylated, were filtered out as to reduce the computational time in the second iteration. At the end of the second iteration the algorithm obtained a *P*-value for each locus. If this *P*-value was smaller than the *P*-value corresponding with an FDR of 0.1, monoallelic methylation on this locus was called significant. This procedure was also performed two times, a first time with 1000 and a second time with 1 000 000 iterations. To summarize results, significant loci were visualized on a circular plot with the Circos tool (33).

### Functional annotation and enrichment analysis

Successful completion of the monoallelically methylated loci detection pipeline resulted in a list of significant SNPs. The functional annotation (i.e. promoter, exon, intron and intergenic) of these SNP positions was determined using Ensembl (release 66), wherein the promoter was defined as starting from 2000 bp upstream until the transcriptional start site.

We tested for enrichment in one or more of these functional categories. A null distribution was generated by random sampling from the total amount of detected SNPs (after filtering as specified in ‘Additional data filtering and correction’ of Materials and Methods) and counting the occurrences of the respective annotations. During this sampling procedure, the number of SNPs sampled for each chromosome was equal to the number of significant SNPs on that chromosome. This sampling was repeated 1000 times. With the null distribution obtained for each of these functional locations (i.e. promoter, exon, intron and intergenic), it was possible to calculate a two-sided *P*-value for each functional location. For loci that were featured by more than one functional annotation (i.e. overlapping genes and/or different transcripts and/or sense and antisense strand) the score for the functional location was divided by the amount of different functional locations that this locus has (the sum always being one). For example, if a locus is located in an exon on the sense strand but is also located in an intron on the other strand, both the exon and intron were attributed a score of 0.5.

### Validation of putative loci using 14 WGBS data sets

In order to evaluate the loci detected by this novel methodology, an extra validation step was performed using 14 publicly available WGBS data sets comprising a range of tissue types. The WGBS data sets were downloaded from the Gene Expression Omnibus repository (34). A summary of the data sets including accession numbers is provided in Supplementary Table S3. The 14 samples were aligned in a window of 2000 bp (1000 bp upstream and 1000 bp downstream) around the candidate SNP positions (hg19/GRCh37 reference assembly) using BISMARK (35). A maximum of three non-bisulfite mismatches was permitted in the seed (70 bp) to (i) lower the presence of sequencing errors while still allowing the detection of real SNPs, but also to (ii) circumvent a possible bias alignment to the reference allele while keeping a substantial unique alignment rate (31). After excluding duplicates, only reads mapping onto one of the candidate monoallelically SNP positions were kept. Next, for each SNP position and each sample the methylation calls, i.e. methylated or unmethylated, of all CpGs were summarized from the mapped bisulfite reads per SNP allele (covered by the reads on the specific SNP position). To assess monoallelic DNA-methylation in the SNP loci a Pearson's chi-square test was performed. With a chi-square test, it could be assessed if each allele has an equal distribution of methylation calls, i.e. degree of methylation. Samples that were not covered or were homozygous for the particular locus were excluded. In summary, for each heterozygous sample a chi-square value was calculated based on the degree of (non-)methylation obtained for each SNP allele, with a high chi-square value indicating that the methylation degree is allele dependent (i.e. monoallelic methylation), i.e. one SNP allele featuring a high degree of methylation while the other allele is characterized with no or a low degree of methylation. Null distributions were made by a permutation approach (using the chisq.test function of the R Stats package) generating 2000 random chi-square values for each sample, making it possible to determine a sample-specific *P*-value for each SNP-loci. By summing the chi-square values over all heterozygous samples for a specific SNP locus and again generating null distributions of random chi-square values, also a global *P*-value for a SNP locus could be obtained. Note that this test does not require absolute absence of methylation of one allele, which would be too strict given the possibility of incomplete bisulfite conversion and the presence of both sequencing errors and sequencing bias.

### Validation of allele-specific expression (ASE) using 16 RNA-seq data sets

As an additional validation and to evaluate the effect of the found monoallelic methylated loci on gene expression, the results were combined with publicly available mRNA-seq data sets from 16 normal human tissues (Illumina's Human BodyMap 2.0 project), including adipose, adrenal, brain, breast, colon, heart, kidney, liver, lung, lymph, ovary, prostate, skeletal muscle, testes, thyroid and leukocyte cells originating from different individuals (15 Caucasians and 1 African American, see Supplementary Table S5). The data are accessible from ArrayExpress, ArrayExpress accession: E-MTAB-513, actual sequence files are in ENA archive with accession number: ERP000546 (linked from ArrayExpress page as ‘ENA - ERP000546’ tag in links section).

For each tissue, the raw paired-end sequence reads (2 × 50 bp) were aligned using the transcriptome mapper STAR (v2.3.1) (36). In order to tackle possible mapping bias to the reference allele, reads were aligned using the method of Degner *et al.* (37), i.e. using the human hg19/GRCh37 as a reference genome which was masked for known dbSNP positions. Reads mapping up to 10 places were allowed with a maximum of 8 mismatches per fragment, i.e. read pair. Only uniquely mapped reads were kept and duplicate fragments were removed with Picard's MarkDuplicates command-line tool (v1.97) (http://picard.sourceforge.net/).

By assessing if some of the found monoallelic methylated loci are associated with ASE, ASE was determined on a per-heterozygote-site per-tissue basis. In a likewise manner as other ASE studies (31,38,39), Samtools mpileup/bcftools (v0.1.19) (40) was used to call possible variants in the non-duplicate, uniquely mapped reads, whereby variant sites with a raw read depth lower than 10 were filtered out and only bases with a minimum base quality (MAQ) of 13 were considered. Next, only SNP positions called by Samtools mpileup and present in dbSNP were kept. Additionally, known dbSNP sites called as homozygous for the reference allele, but where at least two high quality (MAQ ≥ 13) alternate (i.e. non-reference) bases mapped, were also added to the list of variant sites.

After observing the amount of high quality mapped reference versus non-reference bases for each variant site in each tissue, ASE was assessed by performing an exact binomial statistical test with the null hypothesis that each allele is equally expressed. To correct for multiple testing, an FDR of 1% was used. In a next step, the variant sites showing a significant deviation from the binomial distribution were mapped to their corresponding genes. Only genes with at least two significant variant sites were assumed to be allele-specific expressed.

## RESULTS

### Mapping

For the 334 samples the mapping resulted in 2 995 375 490 uniquely mapped reads and an average mapping percentage of 63.05% (Supplementary Table S1). After removing the duplicate fragments a total of 2 688 409 588 non-duplicate, uniquely mapped reads was acquired.

### SNP tracing and data filtering

After parsing the mapping output for SNPs (= mapping mismatches), 19 850 891 SNPs overlapped with already known SNV positions from dbSNP. These 19 850 891 loci represent 41.61% of the total number of SNV present in dbSNP and only these SNPs were used in the remainder of the analysis. Supplementary Table S2 details the number of SNPs that overlapped with dbSNP per chromosome.

After pre-processing the data, the corresponding coverage and allele frequencies were calculated for each of the 19 850 891 loci and subsequently used to filter the data. Only positions with a frequency of the major allele smaller than 0.90 and coverage larger than or equal to 350 (250 for chromosome X) were retained. A total of 486 090 out of 19 850 891 loci (2.45%) complied with these thresholds. Supplementary Table S2 shows the number of SNP positions that were retained after filtering as well as the fraction per chromosome.

### Detection of monoallelically methylated loci

**Figure 2.**
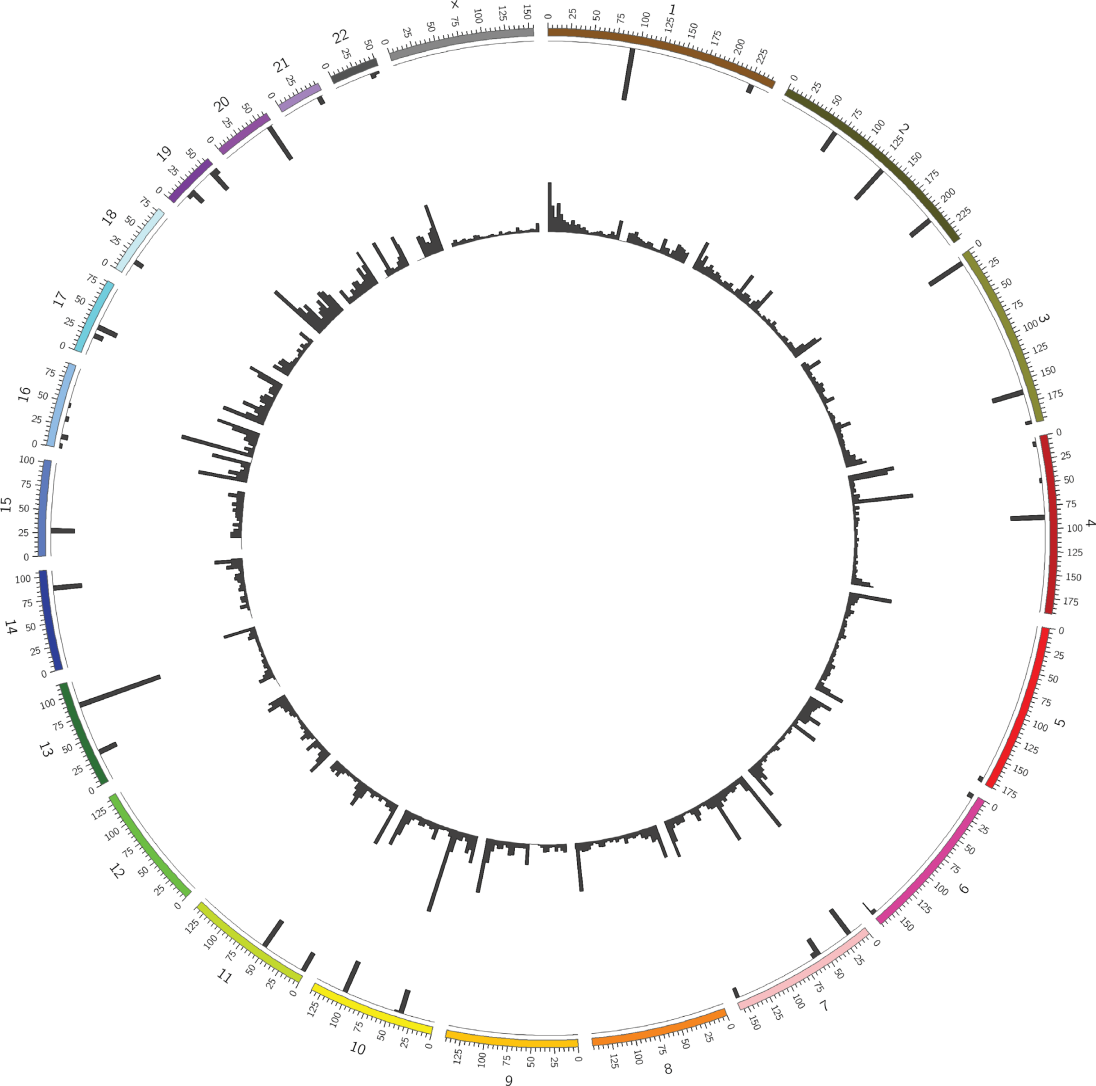
Genomic distribution of the 80 loci for which the monoallelic DNA-methylation was called significant. Chromosomes are shown on a circular representation and divided in regions of 5 000 000 bp. The inner circle shows the histogram of all SNPs found in a specific region, whereas the outer circle shows the histograms of the significant SNPs in that same region, normalized to the number of SNPs found in that region.

Likely sequencing errors in the list of filtered loci were adjusted (see Materials and Methods ‘Additional data filtering and correction’ and Additional Methods Section 2.1.3). Corrected data (available as Additional Data) were subsequently analysed using the developed statistical methodology. If the *P*-value obtained for a locus was smaller than the *P*-value corresponding with an FDR of 0.1 (*P*-value = 0.000016), the monoallelic methylation on this locus was called significant. This was true for 80 loci (see Table 1). Figure 2 depicts the genomic distribution of these 80 monoallelically methylated loci.

**Table 1.** Monoallelic DNA-methylation per chromosome

Chr	Input entries	*P* ≤ 0.005	*P* ≤ 0.000016
1	37 259	98	3
2	34 184	113	10
3	22 065	73	4
4	26 442	68	3
5	20 500	54	1
6	25 708	112	3
7	31 450	153	8
8	20 868	63	0
9	19 908	78	0
10	30 138	108	7
11	19 255	82	8
12	20 484	51	0
13	11 709	88	3
14	12 297	40	1
15	11 829	47	2
16	29 432	64	4
17	24 824	158	3
18	11 783	29	1
19	25 066	96	8
20	16 201	45	7
21	11 977	40	2
22	15 012	70	2
X	7699	27	0
TOTAL	486 090	1757	80

The first two columns show the specific chromosome (Chr) and the number of input entries for the statistical analysis. The third and fourth columns show the amount of loci, which obtained a *P*-value smaller than (or equal to) 0.005 (after first iteration) and 0.000016 (after second iteration, corresponding with FDR = 0.1), respectively.

In a next step, the functional location of the 80 loci with significant monoallelic DNA-methylation was determined. These results are shown in Table 2. Table 3 provides an overview of the genes in which a significant SNP position was found. Thus, of the 80 detected loci, 49 are located in a genic region (i.e. promoter, exon, intron) of which 25 are located in regions with (some) evidence, i.e. monoallelic expression, of imprinting (15,16) (http://www.geneimprint.com).

**Table 2.** SNPs featured by monoallelic methylation located in a gene-associated region

GeneID	Gene symbol	Description	Biotype	Location
ENSG00000183929	*DUSP5P*	Dual specificity phosphatase 5 pseudogene	Pseudogene	1:228757936
ENSG00000200624	*RN5S6*	RNA, 5S ribosomal 6	rRNA	1:228757936
ENSG00000169604	*ANTXR1*	Anthrax toxin receptor 1	Protein coding	2:69347244
ENSG00000233786	*CDC27P1*	Cell division cycle 27 homolog (S.cerevisiae) pseudogene 1	Pseudogene	2:133018988,133020085
ENSG00000163975	*MFI2*	Antigen p97 (melanoma associated)	Protein coding	3:196722009
ENSG00000184985	*SORCS2*	Sortilin-related VPS10 domain containing receptor 2	Protein coding	4:7635629
ENSG00000138641	*HERC3*	Hect domain and RLD 3	Protein coding	4:89618837
ENSG00000177432	NAP1L5*	Nucleosome assembly protein 1-like 5	Protein coding	4:89618837
ENSG00000087116	*ADAMTS2*	ADAM metallopeptidase with thrombospondin type 1 motif, 2	Protein coding	5:178650557
ENSG00000145945	FAM50B*	Family with sequence similarity 50, member B	Protein coding	6:3849305
ENSG00000238158	*RP11-420L9.4.1*	Processed transcript	Processed transcript	6:3849305
ENSG00000184465	*WDR27*	WD repeat domain 27	Protein coding	6:170055316
ENSG00000223838	*AC007091.1.1*	lncRNA	lncRNA	7:19534519
ENSG00000155093	*PTPRN2*	Protein tyrosine phosphatase, receptor type, N polypeptide 2	Protein coding	7:158041459, 158041458, 157923845
ENSG00000075826	*SEC31*	SEC31 homolog B (S.cerevisiae)	Protein coding	10:102279295,102279294
ENSG00000255339	*NDUFB8*	NADH dehydrogenase (ubiquinone) 1 beta subcomplex subunit 8, mitochondrial	Nonsense mediated decay	10:102279295,102279294
ENSG00000166136	*NDUFB8*	NADH dehydrogenase (ubiquinone) 1 beta subcomplex 8, 19kDa	Protein coding	10:102279295,102279294
ENSG00000053918	KCNQ1*	Potassium voltage-gated channel, KGT-like subfamily, member 1	Protein coding	11:2721568
ENSG00000258492	KCNQ10T1*	KCNQ1 opposite strand/antisense transcript 1	Antisense	11:2721568
ENSG00000211502	MIR675**	microRNA 675	miRNA	11:2019496,2019618
ENSG00000130600	H19*	H19, imprinted maternally expressed transcript	Processed transcript	11:2021164, 2019496, 2019618, 2021206, 2021980, 2022023
ENSG00000102802	*C13ORF33*	Chromosome 13 open reading frame 33	Protein coding	13:31481030
ENSG00000226317	*LINC00351*	Long intergenic non-protein coding RNA 351	lncRNA	13:85969909,85969941
ENSG00000258807	*RP11-1152H15.1.1*	lncRNA	lncRNA	14:88237822
ENSG00000214265	SNURF*	SNRPN upstream reading frame	Protein coding	15:25201659
ENSG00000128739	SNRPN*	Small nuclear ribonucleoprotein polypeptide N	Protein coding	15:25201659,25123472
ENSG00000122390	NAA60**	N(alpha)-acetyltransferase 60, NatF catalytic subunit	Protein coding	16:3493495
ENSG00000167981	ZNF597*	Zinc finger protein 597	Protein coding	16:3493495
ENSG00000175643	*RMI2*	RecQ mediated genome instability 2, homolog (S.cerevisiae)	Protein coding	16:11415785
ENSG00000207986	*AC136932.1*	miRNA ncRNA	miRNA	16:33960762
ENSG00000108684	*ACCN1*	Amiloride-sensitive cation channel 1, neuronal	Protein coding	17:31340444
ENSG00000074181	*NOTCH3*	Notch 3	Protein coding	19:15279411
ENSG00000251948	*AC092279.1*	miRNA ncRNA	miRNA	19:24184564
ENSG00000198300	PEG3/ZIM2*	Zinc finger, imprinted 2	Protein coding	19:57350463
ENSG00000259486	ZIM2.1*	Zinc finger, imprinted 2	Protein coding	19:57350463
ENSG00000130844	*ZNF331*	Zinc finger protein 331	Protein coding	19:54057515, 54057777, 54041242, 54057156, 54040861
ENSG00000235590	GNAS-AS1/SANG*	GNAS antisense RNA 1	Antisense	20:57427132, 57414110, 57426449, 57426726
ENSG00000087460	GNAS*	GNAS complex locus	Protein coding	20:57427132, 57414110, 57426449, 57426726, 57431165
ENSG00000160183	*TMPRSS3*	Transmembrane protease, serine 3	Protein coding	21:40757887
ENSG00000182093	*WRB*	Tryptophan rich basic protein	Protein coding	21:40757887
ENSG00000183486	*MX2*	Myxovirus (influenza virus) resistance 2 (mouse)	Protein coding	21:44011806
ENSG00000100138	*NHP2L1*	NHP2 non-histone chromosome protein 2-like 1 (S.cerevisiae)	Protein coding	22:42078666
ENSG00000219438	*FAM19A5*	Family with sequence similarity 19, member A5	Protein coding	22:49077801

Following parameters are indicated: Location (chromosome:location), (Ensembl) Gene ID, Gene symbol, Description and Biotype. *known imprinted gene; **predicted imprinted gene.

**Table 3.** Outcome of the additional validation of the putative loci with 14 WGBS data sets

Chr: SNP position	Global *P*-value	Colon	Colon tumour	Cortex Normal1	Cortex Normal2	Cortex AD1	Cortex AD2	fFF	IMR90	HepG2	HSCP	bcell	dMesenchy	dNPC	dMEEN
1:228757936	0	0.0195	-	0.387	-	0.2695	0	0	0	0	0	0.122	0	0	0
2:133018988	0.248	1	0.5405	0.525	1	1	0.1735	-	-	-	-	-	0.0685	-	0.482
2:133020085	0.0005	0.002	0.7665	0.7875	0.117	0.2655	1	1	1	-	0.029	0.124	0.0925	-	0.3705
2:207122438**	0	0.1345	0.325	0.002	0.4405	-	0.0125	0.3495	0.1685	0.0015	0.5	-	0.03	0.155	0.0015
2:69347244	0.582	-	-	1	-	0.541	-	-	-	-	-	-	-	-	-
2:133033524	0.2535	-	-	-	-	-	-	0.28	-	-	0.6135	-	-	-	-
2:133029769	0.4335	1	-	-	-	-	1	0.175	-	-	-	-	-	-	-
2:133032580	0	0	0	0.7585	0.119	0.5695	0.034	0	0	0	0	0	0	-	0
3:162561619	0	-	-	-	0	-	-	0	-	-	0.0265	-	-	-	0.5525
4:7635629	0	0.0355	-	0.6355	-	-	0	0.004	-	-	-	-	0.00555	-	0.0005
4:89618837*	0	-	-	-	0.0005	0	0.001	0.33	0	-	-	0.0035	0	-	0.0035
4:49099668	0	0.002	0.0015	0.034	0.0755	0.151	0	0	0.0595	0.0015	0.004	0.3295	0.089	-	-
5:178650557	0	-	-	-	0	-	-	-	-	-	0.3215	-	0.0045	0.226	-
6:170055316	0	-	-	0	-	-	-	0.002	-	0.2125	0.4965	0.566	0	-	0.2345
6:3849305*	0	0	0	0	0.0065	0	0.0025	0.6925	0.48	0.0025	-	0	1	0.1505	-
6:168784228	0	0	0.04	-	-	0.0315	0	0.0005	0.0025	0	-	-	0.003	-	-
7:64895556	0.0265	-	0.38	-	-	-	1	0.0005	-	-	-	-	-	0.075	-
7:157923845	0.3995	-	-	-	-	-	-	0.3995	-	-	-	-	-	-	-
7:61080848	1	-	-	-	-	-	-	-	-	-	1	1	-	-	-
7:56437045	0	-	-	0	-	-	-	0	-	-	-	-	-	0.001	0.0775
7:19534519	0.307	-	-	-	-	-	-	1	-	-	0.3905	-	-	-	0.33
7:57554497	0.0205	-	-	-	-	-	-	0.0275	-	-	0.269	-	-	-	0.574
10:42800026	0.5405	-	-	-	-	-	-	1	-	-	-	1	-	-	0.27
11:2721568*	0	0	0	0	0	-	0	0	-	-	0	0.1275	-	-	-
11:51579458	0.5285	-	-	-	-	-	-	0.5285	-	-	-	-	-	-	-
13:31481030	0.096	0.1905	-	-	-	-	-	-	0.174	-	-	-	-	-	-
14:88237822	0.17	0.184	-	-	0.2815	1	-	0.1725	-	-	-	0.0445	-	-	0.6605
15:25123472*	0	0	0	0.0475	0.003	0	0	0.4215	0.314	-	0.0045	0	-	-	-
15:25201659*	0	-	-	0	0	-	-	0.5625	-	-	0.4805	0.012	0	0	0
16:46411729	0	1	0.6475	-	-	-	-	0	-	0.546	-	-	-	-	-
16:3493495*	0	-	-	0	0	-	0.0355	0	0	-	0.861	0.0095	0	-	0.001
16:11415785	0.002	-	-	-	-	-	-	0.0475	0	-	-	-	-	-	-
17:22252007	0.74	1	-	0.6095	-	-	0.627	-	-	-	0.6155	-	-	-	-
17:22259640	0.7215	0.8215	0.7795	0.3425	1	1	0.613	0.3785	0.6265	-	0.212	0.7165	1	-	-
18:18517029	0	-	0.0415	-	0.017	0.1795	0.0045	0	-	-	0	0.307	-	-	0
19:15279411	0	-	-	0.299	0.054	0	0.0005	0	-	0.568	0.839	0.0155	0	-	0.016
19:57350463*	0	0	0.0005	0	0	0	-	0.004	-	0	0.2945	0.0095	0	0	0
19:24184564	0	-	-	-	-	-	-	1	-	-	-	-	-	0	0.0005
20:57415110*	0	-	-	0.016	0	-	0	0	0.0095	0.4675	-	0	0	-	0
20:57431165*	0.0625	-	-	0.5545	-	-	-	0.011	0.8245	-	-	-	-	-	-
21:44011806	0.0145	-	-	-	-	-	-	0.0145	-	-	-	-	-	-	-
21:40757887	0	0	0	-	-	-	-	0.0255	-	-	0	-	1	-	-
22:42078666	0	0	0.005	-	0	-	-	-	-	-	-	-	0	-	0.013
22:49077801	0.715	-	-	-	-	-	-	0.715	-	-	-	-	-	-	-

Global and sample-specific *P*-values are shown for the 44 SNP loci (Chr:SNP location) that were covered by at least one heterozygous WGBS sample. Value ‘-’ in the sample columns indicates that the sample did not cover or was not heterozygous for the corresponding SNP loci. *known imprinted genomic region; **predicted imprinted genomic region. Samples: colon adjacent normal (Colon), colon primary tumour (Colon tumour), mid frontal cortex normal (Cortex Normal1/2), mid frontal cortex Alzheimer (Cortex AD1/2), newborn foreskin fibroblasts (fFF), human foetal lung cell line (IMR90), human liver carcinoma cell line (HepG2), hematopoietic stem cell progenitors (HSCP), human B cells (bcell), H1-derived mesenchymal stem cells (dMesenchy), H1-derived neuronal progenitor cells (dNPC) and H1+BPM4-derived mesendoderm cells (dMEEN).

### Functional enrichment of loci with significant monoallelic DNA-methylation

**Figure 3.**
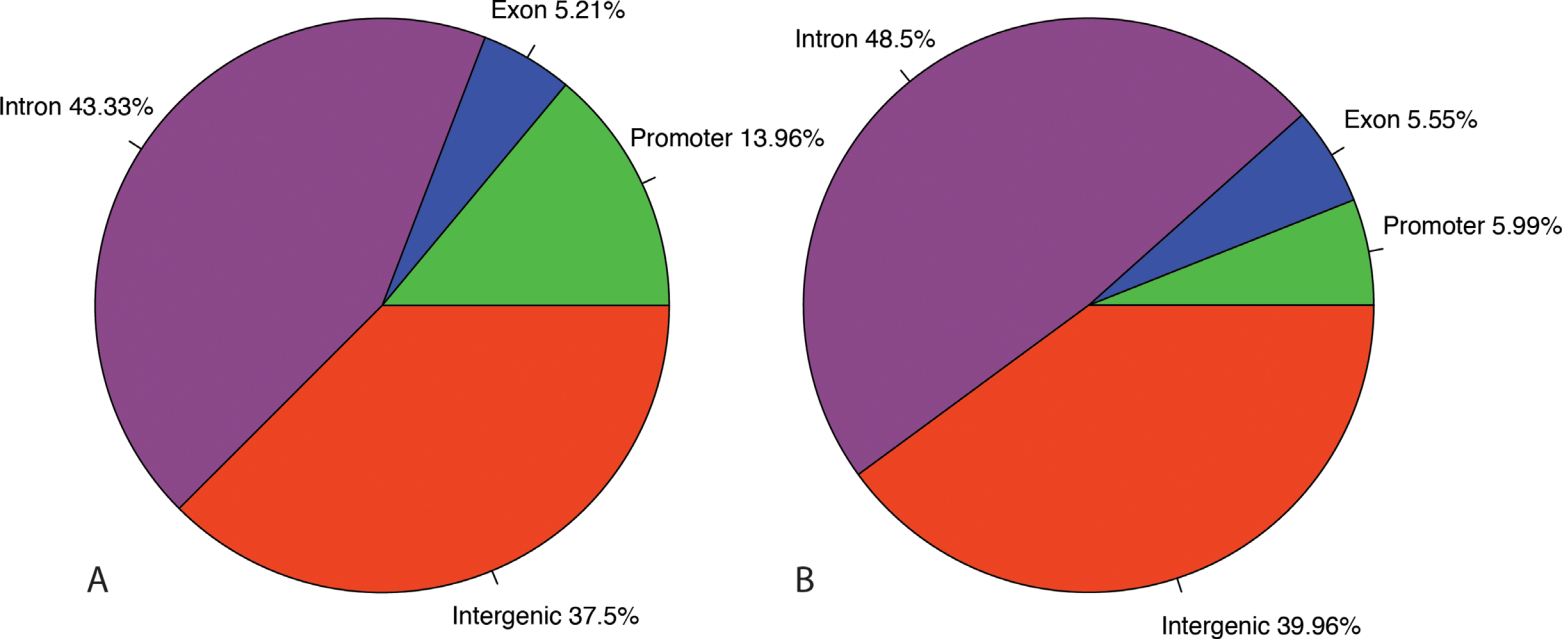
Pie charts representing the relative number of significant SNPs in the different functional classes. (A) Functional classification (i.e. promoter, exon, intron and intergenic) of the significant SNPs (i.e. loci with significant monoallelic DNA-methylation). (B) Functional classification of random SNPs resulting from 1000 iterations.

Figure 3(A) represents the relative number of the different functional annotations of these 80 loci. No significant enrichment was found when genic regions were compared to intergenic regions (data not shown). The majority of the significant SNP positions are located in intronic (43.33%) and intergenic regions (37.5%). Additionally, a significant number was found in the promoter regions (13.96%). A minority of 5.21% mapped to exonic regions. In order to investigate whether one of these functional locations was under- or overrepresented compared to random data, we also performed an enrichment analysis. Figure 3(B) shows the mean classification of SNPs after 1000 random samplings. By comparing the outcome of this random sampling with the functional locations of the 80 significant loci (see Materials and Methods ‘Functional annotation and enrichment analysis’), the analysis indicated a significant enrichment in promoter methylation (*P*-value = 0.002), but not in other functional locations.

### Validation of putative loci using 14 WGBS data sets

After pre-processing the 14 WGBS data sets as outlined in Materials and Methods ‘Validation of putative loci using 14 WGBS data sets’, 44 out of the 80 significant loci were covered by at least one heterozygous sample. Table 4 summarizes both the global and the sample-specific *P*-values obtained for each of these 44 loci. Note that 29 loci (65.9%) had a global *P*-value lower than 0.05 of which 24 (54.5%) even had a global *P*-value virtually equal to 0 suggesting monoallelic methylation in at least one of the 14 samples.

**Table 4.** Validation of ASE

GeneID	Gene symbol	# Tissues	Tissues	Type of expression
ENSG00000219438	*FAM19A5*	1	testis	ASE
ENSG00000184985	*SORCS2*	1	brain	ASE
ENSG00000169604	*ANTXR1*	1	adipose	ASE
ENSG00000255339	*NDUFB8*	1	heart	ASE
ENSG00000166136	*NDUFB8*	10	adipose, adrenal, brain, breast, colon, heart, ovary, prostate, testis, thyroid	ASE
ENSG00000130600	H19*	10	adipose, adrenal, breast, colon, kidney, ovary, prostate, skeletal muscle, testis, thyroid	ASE
ENSG00000175643	*RMI2*	1	testis	ASE
		1	ovary	BE
ENSG00000122390	NAA60**	1	brain	ASE
		10	adipose, adrenal, breast, colon, heart, kidney, liver, lymph node, ovary, thyroid	BE
ENSG00000138641	*HERC3*	1	brain	ASE
		4	heart, leukocyte, ovary, prostate	BE
ENSG00000155093	*PTPRN2*	1	brain	ASE
		1	prostate	BE
ENSG00000182093	*WRB*	2	brain, liver	ASE
		7	adrenal, heart, leukocyte, ovary, skeletal muscle, testis, thyroid	BE
ENSG00000198300	PEG3/ZIM2*	2	brain, ovary	ASE
		1	testis	BE
ENSG00000183486	*MX2*	2	adipose, leukocyte	ASE
		4	breast, ovary, testis, thyroid	BE
ENSG00000130844	*ZNF331*	2	brain, ovary	ASE
		1	lung	BE
ENSG00000074181	*NOTCH3*	4	adipose, adrenal, breast, testis	ASE
		6	colon, heart, lymph node, ovary, skeletal muscle, thyroid	BE
ENSG00000214265	SNURF*	4	brain, lymph node, prostate, testis	ASE
		2	heart, thyroid	BE
ENSG00000128739	SNRPN*	7	adrenal, colon, leukocyte, lymph node, ovary, prostate, testis	ASE
		1	brain	BE
ENSG00000100138	*NHP2L1*	8	adrenal, brain, heart, kidney, leukocyte, ovary, prostate, testis	ASE
		7	adipose, breast, colon, liver, lung, lymph node, thyroid	BE
ENSG00000087460	GNAS*	13	adipose, adrenal, brain, breast, heart, kidney, leukocyte, lung, lymph node, ovary, prostate, testis, thyroid	ASE
		1	colon	BE
ENSG00000235590	GNAS-AS1/ SANG*	1	testis	BE
ENSG00000087116	*ADAMTS2*	3	adipose, breast, ovary	BE

Results are shown for the 21 genes with one (or more) monoallelic methylated SNP(s) in their genic regions and reached the thresholds to investigate putative ASE. Six genes exclusively show ASE in one or multiple tissues, 13 genes have both ASE and biallelic expression (BE) in different tissues and 2 genes only show BE. Following columns are indicated: (Ensembl) Gene ID, Gene symbol, number and annotation of tissues for which ASE/BE could be examined (# Tissues and Tissues, respectively) and the Type of expression found for these tissues (ASE or BE). *known imprinted genomic region; **predicted imprinted genomic region.

### Validation of ASE using 16 RNA-seq data sets

To validate if the found monoallelically methylated loci are associated with ASE, publicly available mRNA-seq data sets from 16 different individuals and tissues were searched for ASE. After pre-processing the data for each variant site, binomial tests were performed. Using an FDR of 1%, and requiring the presence of at least two significant variant sites per gene, in total, 19 840 genes showed ASE, ∼1190 genes per tissue (Supplementary Table S5), in line with the amount of loci identified in previous studies (1,41–43).

In a next step, it was examined if some genes from Table 2—the genes with one (or more) monoallelic methylated SNP(s) in their genic regions—were also characterized by ASE. Indeed, 21 of the 43 genes were covered by at least two variant sites in some tissues, of which 19 showed ASE in one or more tissues (Table 4). Of these 19 genes, 13 also showed biallelic expression in other tissues. For the remaining two genes, *GNAS-AS1* and *ADAMTS2*—although covered by two or more variant sites in some tissues—ASE could not be validated and thus showed evidence of biallelic expression. Of the 19 allele-specific expressed genes, 6 have already been linked to imprinting (15,16) (http://www.geneimprint.com). The other 13 genes represent novel candidate imprinted genes. In addition, for *WRB*,*NHP2L1*,*NAA60*,*ZNF331*,*H19* and *GNAS* the found monoallelic methylated loci were located in their respective promoter region. Supplementary Table S6 also lists the results for genes called allele-specific expressed if at least one significant variant site was present.

## DISCUSSION

Monoallelic gene expression is typically initiated early in the development of an organism and stably maintained. Erroneous monoallelic expression has been related to several non-Mendelian inherited genetic disorders. DNA-methylation plays a significant role in the regulation of monoallelic expression. The choice of the allele to be monoallelically expressed can be either random or *a priori* defined by imprinting. Here we introduced a methodology to screen for genes that exhibit monoallelic DNA-methylation and thus might regulate monoallelic expression.

Using MethylCap-seq, methylome profiles of 334 samples, mostly human cancer samples of diverse origin, were obtained. In summary, upon extra filtering and data correction, for each SNP locus the Hardy–Weinberg theorem was applied to evaluate whether the observed frequency of samples featured by biallelic methylation is lower than randomly expected. Using a permutation approach, loci with a *P*-value smaller than the *P*-value corresponding with a selected FDR of 0.1 were assumed to be monoallelically methylated. Finally, this resulted in the identification of 80 loci that showed significant monoallelic DNA-methylation.

Functional location of these monoallelic events might provide deeper insight in the unraveling of monoallelic mechanisms and are provided in Table 2 and Supplementary Table S4. It is common that imprinted genes are present within clusters and share common regulatory elements, such as non-coding RNAs and differentially methylated regions (DMRs). If these DMRs control the imprinting of one or more genes, these regions are called imprinting control regions (ICRs). It is known that many of these ICRs are located in intergenic regions. As some of the found loci are located in intergenic regions as well as in known (long) non-coding RNAs (lncRNAs) (see Table 2 and Supplementary Table S4, respectively), it is possible that these regions present new regulatory elements involved in imprinting. Furthermore, when we take a closer look at the intergenic regions, the SNP on chromosome 2 with position 207 122 438 also shows significant monoallelic methylation. This is interesting, because this locus falls in *GPR1AS*, a recently found imprinted lncRNA in the *GPR1-ZDBF2* intergenic region (44), corroborating the outcome of this study and indicating that the so-called ‘intergenic’ regions are also of interest for further analyses. Of the 80 loci, 49 were located in genic regions of which 25 are already linked to imprinting. For example, on chromosome 11 (2.01–2.03 mega base (Mb)), the *IGF2/H19* region was highlighted with 6 SNPs (Figure 2). This locus is a well-known imprinted region that is also linked to the Beckwith–Wiedemann syndrome and Wilms’ tumour (4,45–47). The *H19* gene codes for a lncRNA of which expression is negatively correlated with the expression of the neighbouring gene insulin-like growth factor 2 (*IGF2*). Usually the paternal copy of *H19* is methylated and silent, while the maternal copy is hypo- or unmethylated and expressed. The same is true for the imprinted region on chromosome 15 that is correlated to the Prader–Willi syndrome (20.7–30.3 Mb) (4).

In *SNRPN*, one of the genes in this region where loss of imprinting is linked to the Prader–Willi syndrome, 2 significant SNPs were identified. For a couple of genes (or regions), like, for example, *H19*, more than one significant SNP locus was found. Because some of these SNPs are at a distance of more than 400 bp (the cut-off length of sequence reads during mapping) of each other, these prove independently the presence of monoallelic DNA-methylation in that particular region. These SNPs thus provide ‘multiple proof’ in the identification of the particular monoallelically methylated region and lend added value to the results. Not unexpectedly, Figure 3 and the enrichment analysis clearly demonstrated enrichment for monoallelic methylation in promoter regions, although it should be noted that this enrichment is rather limited in absolute number.

In females, most of the genes on one X-chromosome are transcriptionally silenced by monoallelic epigenetic events like DNA-methylation and histone modifications. Early in development, each cell makes an independent, random choice which chromosome to inactivate. Once this decision is set, all further descendants of that cell keep the same pattern. As our method is designed to specifically detect a deviation from the Hardy–Weinberg equilibrium, it is necessary that for one sample, the same allele is (un)methylated for all cells—and thus not randomly chosen. In summary, as random monoallelic methylation would lead to the detection of more heterozygotes, the fact that no monoallelic methylated loci were found on the X-chromosome reassures the detection of stable monoallelic methylation with our method.

Further validation was performed using 14 publicly available WGBS data sets, comprising 10 normal samples and 4 samples derived from non-normal tissue, including two samples of cancerous origin (colon tumour tissue and a human liver carcinoma cell line) and two brain samples from Alzheimer patients. Of the 80 significant loci, 44 were covered by a heterozygous sample and could thus be further examined. Twenty-nine of the 44 loci (65.9%) obtained a global *P*-value lower than 0.05 of which 24 (54.5%) had a global *P*-value of virtually 0, indicating monoallelic methylation in one or more samples. For only 9 of these 24 SNP loci evidence of imprinting already exists, so that with this subset of 14 WGBS samples at least 15 new monoallelically methylated regions, found with our new data-analytical framework, are validated. Furthermore, from the sample-specific *P*-values in Table 3 it can be seen that these 24 loci are mainly validated in samples of normal origin, whereby each locus is validated in multiple normal samples—and thus not or not only in cancerous/diseased samples.

In addition, with the ASE analysis of 16 different tissues of normal origin it was possible to investigate if (some of) the found monoallelically methylated regions are associated with ASE—and thus might regulate this monoallelic expression. Of the 43 genes featured by one or more of the detected monoallelic methylated loci, 21 had variants with sufficient coverage and base quality, and were further examined. Of these, 13 genes showed both allele-specific as well as biallelic expression in different tissues, while 6 genes were only featured by ASE (Table 4). The remaining two genes could not be validated as allele-specific expressed in a tissue. Of the 19 genes featured by ASE, only 6 have already been linked to imprinting, suggesting the identification of novel candidate imprinted genes. In fact, for two of these ‘novel’ imprinted genes, *WRB* and *NHP2L1*, recent evidence by Docherty *et al.* strongly suggests that these are indeed putatively imprinted as (i) they show ASE in some tissues and (ii) their methylation patterns are consistent with allelic maternal methylation (48). Furthermore, for *WRB* and *NHP2L1* as well as *NAA60*,*ZNF331*,*H19* and *GNAS* monoallelic promoter methylation was found.

There are a couple of important remarks that come with the proposed methodology:

- The basic assumption that MethylCap-seq data from biallelically methylated loci are generally in Hardy–Weinberg equilibrium only holds for samples originating from a panmictic population (i.e. a single population that is long-term randomly mating). If this is not the case and the samples are not panmictic, this could possibly give rise to some false positives. Thus, for samples that slightly deviate from the assumption of panmixia, an extra validation of the resulting loci is necessary to assure qualitative results (as was done in this study).
- The approach does not take into account that loci with monoallelic methylation will be picked up less efficiently than biallelic loci resulting in less power leading to a less efficient detection of monoallelically methylated loci. By consequence, the methodology is less sensitive and thus too conservative, though this has no effect on the reliability of those results deemed significant.
- It is known that aligning to a reference genome at sites of DNA-variants generates a bias towards higher mapping rates of the reference allele compared with the alternative allele (37). Recently, Stevenson *et al.* (31) showed that increasing the number of mismatches significantly improved measures of allelic abundance, and demonstrated that a maximum of three mismatches provides a good trade-off, as implemented in this study. Also, as the SNP density in the human genome is approximately one SNP per kilobase (49), this trade-off is deemed to be sufficient to both allow the occurrence of real SNPs as well as to lower the presence of possible sequencing errors. By not calculating the observed allele frequencies from sample-specific allelic abundances but from the observed genotypes (see Additional Methods), the possible influence of an allelic bias is further minimized. In conclusion, these precautions together with the fact that our method is conservative ensure that our method is not affected by a possible bias alignment. Indeed, an extra quality control of the monoallelically methylated loci showed no difference in sample coverage between samples homozygous for the reference and the alternative allele (Wilcoxon rank-sum test, *P*-value = 0.21, data not shown).
- To eliminate sequencing errors as well as to reduce the computational time and effort a filtering step was performed. Consequently, some data will not be analysed and this could interfere with the detection of monoallelic DNA-methylation. However, the benefits of filtering outweigh the possible drawbacks: the computational load reduces significantly and the power to detect loci that do not pass the filter cut-off will be typically insufficient.
- The approach used to correct for possible sequencing errors disfavours the presence of monoallelic DNA-methylation: only two correction rounds were performed and the sequencing error estimate of 0.25% is the lowest estimate reported (32). But although the correction method can be considered a bit too stringent, it will assure a better quality of the obtained results and will not give rise to more false positives. In fact, it will possibly reduce the amount of false negatives and thus allows a more sensitive identification of monoallelically methylated loci that would otherwise not have been detected.
- The analysis was performed with samples originating from different tissues that were mostly cancer tumours. The fact that tumours are epigenetically less stable than healthy tissue (50), makes it probably more difficult to detect monoallelic methylation. On the other hand, it is known that chromosomal deletions and loss of heterozygosity frequently happen in cancer, both leading to possible ‘monoallelic’ methylation events. However, as a mixture of different cancer tumours was used and it is very unlikely that these are all characterized by the same chromosomal deletion, this latter phenomenon will have had little effect on our stringent analysis. To justify this, additional analyses were performed on 14 WGBS data sets (of which 10 were of normal origin) as well as on mRNA-seq data of 16 tissues of normal origin, validating a notable number of the identified loci and detecting putatively imprinted genes.

Although the employed experimental set-up to test our methodology is somewhat challenging—using a mixture of samples originating from different tissues, mostly cancer tumours—the proposed methodology allowed the identification of loci known to be generally imprinted and involved in genetic and/or imprinting disorders (e.g. *IGF2*/*H19*, *KCNQ10T1*, *SNURF*/*SNRPN*, *GNAS*, …) demonstrating the robustness and biological relevance of our method. Additionally, the extra ASE analysis identified monoallelic methylated loci associated with ASE, thereby identifying 6 known and 13 novel candidate imprinted genes (e.g. *WRB*,*NHP2L1*,*…*). As we opted to use a stringent approach, the outcome further demonstrates that our methodology is still sensitive enough and produces satisfying results.

As recent evidence suggests that monoallelic DNA-methylation is often tissue- or cell-type specific (19,51), it would be particularly interesting to apply the methodology on MethylCap-seq samples of normal, single-tissue origin, ideally from a single population. Next to MethylCap-seq, our approach also opens the door to other applications, like ChIP-seq-based detection of monoallelic protein-DNA binding events and histone modifications.

## AVAILABILITY

Additional Data (filtered and sequence error corrected SNP-loci of the 334 samples, i.e. starting data of the data-analytical framework) as well as corresponding scripts of the developed statistical methodology are available on the authors website: http://www.biobix.be/MAM/.

## SUPPLEMENTARY DATA

Supplementary Data are available at NAR Online.

SUPPLEMENTARY DATA
