# Supplementary material for: SNP-guided identification of monoallelic DNA-methylation events from enrichment-based sequencing data
Source: Nucleic Acids Res. 2014 Sep 18;42(20):e157. doi: 10.1093/nar/gku847 (PMC4227762; doi:10.1093/nar/gku847)
Supplement: SUPPLEMENTARY DATA [file supp_42_20_e157__index.html]

SNP-guided identification of monoallelic DNA-methylation events from enrichment-based sequencing data — SNP-guided identification of monoallelic DNA-methylation events from enrichment-based sequencing data — SUPPLEMENTARY DATA 

# SNP-guided identification of monoallelic DNA-methylation events from enrichment-based sequencing data

## SUPPLEMENTARY DATA

**Files in this Data Supplement:**

- SUPPLEMENTARY DATA
