# Supplementary material for: SNP-guided identification of monoallelic DNA-methylation events from enrichment-based sequencing data
Source: Nucleic Acids Res. 2014 Sep 18;42(20):e157. doi: 10.1093/nar/gku847 (PMC4227762; doi:10.1093/nar/gku847)
Supplement: SUPPLEMENTARY DATA [file supp_gku847_nar-01370-met-g-2014-File005.zip › SupplementaryData/AdditionalMethods.pdf]

SNP-guided identification of monoallelic DNA-methylation events from enrichment-based sequencing data

Sandra A. Steyaert[1], Wim Van Criekinge[1], Ayla De Paepe[1], Simon Denil[1], Klaas Mensaert[1], Katrien Vandepitte[2], Wim Vanden Berghe[3], Geert Trooskens[1] and Tim De Meyer[1]

[1] *Department of Mathematical Modelling, Statistics and Bioinformatics, University of Ghent, Coupure Links 653, 9000 Ghent, Belgium*

[2] *Department of Biology, University of Leuven, Naamsestraat 61 box 2464, 3000 Leuven, Belgium*

[3] *PPES, Department of Biomedical Sciences, University of Antwerp, Universiteitsplein 1, 2610 Wilrijk, Belgium*

ADDITIONAL METHODS

1 Additional Methods

Here we present the methodology of the data-analytical framework to screen for monoallelic DNA-methylation in sequencing data obtained from MethylCap-seq. In summary, MethylCap-seq, which combines precipitation and thereby enrichment of methylated DNA-fragments by a recombinant methyl-binding domain (MBD) with massively parallel sequencing of the isolated DNA, was used to profile the DNA-methylation pattern of 334 human samples, mostly cancer samples. Preliminary to the sequencing step, the DNA was fragmented to pieces of approximately 200 nucleotides making use of a Covaris S2 instrument. The resulting fragments of the different samples were subsequently multiplexed, followed by paired-end sequencing on the Illumina Genome Analyser IIx (GAIIx) or the Illumina HiSeq. Depending on the sequencing platform, the resulting reads had a length of 45 and 50 bp, respectively. Upon mapping of sequenced reads and single nucleotide polymorphism (SNP) identification, loci were filtered based on major allele frequency and coverage, and additional data correction for sequencing errors was performed. Subsequently, the SNP profiles were combined with the methylome profiles to detect regions that display monoallelic DNA-methylation. In the next sections, the global idea and strategy of both the additional sequencing error correction and the data-analytical framework will be discussed in detail. The developed approach will also be illustrated with two hypothetical examples.

1.1 Data-analytical framework: general discussion

Besides the description of the data preprocessing (section 1.1.1), the different steps of the statistical methodology (sections 1.1.2 and 1.1.4) and the procedure followed to correct for possible sequencing errors (section 1.1.3), a global overview as well as a discussion of the practical implementation will also be presented (section 1.1.5).

1.1.1 Data preprocessing: mapping and SNP identification

The paired-end reads were mapped using BOWTIE (30) to the human reference genome (NCBI build 37). During the mapping process, no more than three mismatches were allowed. Based on the SNP database of NCBI (dbSNP, version 137), in the obtained non-duplicate, uniquely mappable sequence reads the SNPs were tracked down so that, in addition to the methylome profile, also the SNP profile of the methylated fragments became available for each sample. Sequencing errors are characterized by their low frequency of appearance, i.e. a very low frequency of the 'minor allele' (= high frequency of the major allele). Both for computational reasons and to remove possible sequencing errors from the found SNPs, it was opted to filter the data based on a predetermined maximal frequency for the major allele (0.9) as well as a minimal total coverage threshold (250 for the X-chromosome, 350 for other chromosomes $\sim$ number of analysed samples). Note that real SNPs with such a low allele frequency and/or coverage contribute very little to the identification of monoallelically methylated loci (too low power). As a second filter for sequencing errors, an additional data correction was performed (cf. infra section 1.1.3).

1.1.2 Estimation of the allele and genotype frequencies

Firstly, by parsing the mapping output and using dbSNP, the obtained MethylCap-seq data is being screened for SNPs. These SNPs make it possible to discriminate between homo- and heterozygous samples for each locus. Suppose there are S samples with L loci in which SNPs were found. For each sample s (1 to S) and for each locus l (1 to L) n_{sl} sequences are observed with $\mathrm{n}_{sl} \geq 0$. In these, there are $\mathrm{n}_{sl,t}$ sequences with a thymine, $\mathrm{n}_{sl,c}$ sequences with a cytosine, $\mathrm{n}_{sl,a}$ sequences with an adenine and $\mathrm{n}_{sl,g}$ sequences with a guanine for the relevant SNP position (= locus), such that consequently $\mathrm{n}_{sl,t} + \mathrm{n}_{sl,c} + \mathrm{n}_{sl,a} + \mathrm{n}_{sl,g} = \mathrm{n}_{sl}$. As the human genome is diploid, at least two of these numbers are zero, with the possible exception of sequencing errors. If for a locus both alleles are observed in a sample, then this sample is called heterozygous. If on the other hand only one allele is observed, this sample is called homozygous for this locus. From these **observed genotypes**, the **allele frequencies** can be estimated.

The estimation of these frequencies might however be complicated by a limited coverage for a specific locus, resulting in heterozygous samples appearing as putative homozygous samples. When for example both allele A and allele B are present, and both alleles are methylated, it might occur that only allele A will be observed due to limited coverage for this locus. However, ignoring the putatively very limited effect of sequencing bias on SNP coverage, the observation of a specific allele can be considered to be the result of a random sampling process. Therefore, with a sufficient amount of samples, errors will be averaged yielding an unbiased estimate of the allele frequencies. For example, assume that there are 20 heterozygous samples wherein 10 of the samples the coverage was too low to observe both alleles, A and B. Because every allele has an equal chance of being picked up, it is expected that in 5 cases only A and in the other 5 cases only B will be observed.

If in each case these samples are considered to be homozygous, the **estimated allele frequency** (10 AB + 5 AA + 5 BB = 20 A and 20 B) is equal to the actual allele frequency (20 AB = 20 A and 20 B). When sequencing errors are not taken into account, real homozygotes will always be observed as homozygotes and thus the estimation remains correct. The possible extension to three or four alleles in the population doesn't have a further impact on the determination of the allele frequencies.

The remainder of this analysis will work with the **observed fraction of heterozygotes**. Under the null hypothesis, i.e. no presence of monoallelic methylation, using the Hardy-Weinberg theorem (22), it is possible to calculate the expected genotype frequencies. For the homozygous fractions TT, AA, CC and GG the resulting **theoretically expected genotype frequencies** (i.e. the expected genotype frequencies under the null hypothesis) are then $(n_{sl,t}/n_{sl})^2$, $(n_{sl,a}/n_{sl})^2$, $(n_{sl,c}/n_{sl})^2$ and $(n_{sl,g}/n_{sl})^2$, respectively, while for the different x,y heterozygotes this gives $2*(n_{sl,x}*n_{sl,y})/(n_{sl})^2$.

1.1.3 Additional data correction

Candidate SNPs identified in section 1.1.2 are either real SNPs or the result of sequencing errors. An additional data correction step was performed to reduce noise caused by possible sequencing errors. This correction makes sure that for every individual sample there are at most two values of $n_{sl,t}$, $n_{sl,c}$, $n_{sl,a}$ and $n_{sl,g}$ bigger than zero (see section 1.1.2). For computational reasons a heuristic approach was chosen rather than an advanced analysis of the fastq quality scores. Remaining variation in the sequence reads, like for example indels, usually results in the inability to map these reads on the reference genome using BOWTIE, so that this kind of variation will not be taken into account.

For a particular locus *l* and a particular sample *s* the obtained sequencing data can have a similar format like one of the following three questionable situations:

- Situation 1: AAAAAAAAAAAAAAAT
  In this situation, the data consists of two alleles but one allele is overrepresented (A). Although the determination of the genotype will result in a heterozygote (AT), this was most likely a homozygous sample for allele A where allele T was probably the result of a sequencing error. To correct for these 'false' heterozygotes, for every heterozygous sample two probabilities are being calculated: 1. the probability that this sequence list is the result of a homozygote (ho) in which there is a sequencing error (se) present ($P_{ho\&se}$) and 2. the possibility that this sequence list is indeed the result of a heterozygous (he) sample (sa) ($P_{he\&sa}$).

  The calculation of $P_{ho\&se}$ and $P_{he\&sa}$ goes as follows:

  - $P_{ho\&se} = P_{se|ho} * P_{ho}$
    Where:

  * $P_{se|ho}$ = the probability of success (i.e. that you observe this sequence) in a Bernouilli experiment given the coverage of the allele with the lowest frequency (= number of successes, here: coverage of allele T), the sample coverage (= number of trials, here: coverage of allele A + coverage of allele T) and the possibility of a sequencing error (= hypothesized probability of success). The sequencing error rate was set to the most conservative (= lowest) value found in literature, namely 0.25% (32).
  * P_{ho} = the probability of having a homozygote in the population (i.e. all samples), given the population frequency of the most frequent allele in the sample (= $freq_{most}$, here: frequency of allele A). Thus, $P_{ho} = (freq_{most})^2$.
- $P_{he\&sa} = P_{sa|he} * P_{he}$
  Where:
  * $P_{sa|he}$ = the probability of success (i.e. that you observe this sequence) in a Bernouilli experiment given the coverage of the allele with the lowest frequency (= number of successes, here: coverage of allele T), the sample coverage (= number of trials, here: coverage of allele A + coverage of allele T) and finally the possibility of having a heterozygous sample (= hypothesized probability of success) which was assumed to be 50%.
  * P_{he} = the probability of having a heterozygote in the population (i.e. all samples), given the frequencies of both alleles (= $freq_1$ and $freq_2$, here: frequencies of alleles A and T) in the whole population. Thus, $P_{he} = 2*freq_1*freq_2$.

If $P_{ho\&se}$ is bigger than $P_{he\&sa}$ the putative sequencing error will be removed and the data is corrected to AAAAAAAAAAAAAAA.

- Situation 2: AAAAAATTTTTTC
  Although this data is obtained from a diploid sample, the resulting genotype is a triploid. Intuitively, the genotype would be AT, so that also here one detected allele (C) is due to a sequencing error. These situations are adjusted by removing the nucleotides of the allele with the lowest coverage in the sample from the obtained sequencing data. Here, this results in AAAAAATTTTTT and now consists only of two alleles instead of three. The corresponding genotype is AT. As this is now a (diploid) heterozygote, in a next step, $P_{ho\&se}$ and $P_{he\&sa}$ are also determined for this sequence using the same procedure as the one described in situation 1.

- Situation 3: AAAATC
  In this situation, also three different alleles are picked up, but here the two alleles with the lowest frequencies (T and C) have an equal coverage. The correction method for this situation is thus less straightforward than the triploid of situation 2. Nevertheless, these kinds of dubious situations can be corrected in a likewise manner as for situation 2: remove the allele, which has the lowest allele frequency throughout all the samples (i.e. the population

frequency). The resulting data will now consist of two alleles and again will go through the same procedure as in situation 1.

Because after each correction round the allele frequencies of the population are updated, an iterative approach was used. Due to the computational load, this correction procedure of the data was only performed twice, but in most cases this was already sufficient to obtain stable results. It should also be noted that this procedure is relatively conservative, i.e. the observed fraction of heterzygotes will likely be overestimated.

1.1.4 Identification of loci with significant monoallelic methylation

Taken altogether, the identification of SNP loci with significant monoallelic methylation results in finding a significant discrepancy between the **observed** and the **theoretically expected heterozygous fractions**. Indeed, in case of perfect monoallelic methylation, the **observed heterozygous fraction** equals zero.

However, due to the low coverage that can occur for some loci, there can be a bias in the **observed fraction of heterozygotes**: due to stochastic effects, only a single allele might be captured from biallelically methylated samples, resulting in the latter to be observed as homozygous. Note that this effect does not affect the estimation of the allele frequencies (cf. supra section 1.1.2). In order to correct for this bias an iterative sampling procedure is used, where data is simulated under the null hypothesis for the locus *l* under the study. Practically, S genotypes are sampled from the total pool of possible genotypes with probability weights equal to the **theoretically expected genotypes** for *l*. Subsequently, the S observed coverages for locus *l* are permuted and attributed to the S created genotypes. For each created heterozygous genotype, an amount of alleles equal to the attributed coverage is sampled from both alleles (with equal chance). This sampling procedure will result in a lower amount of heterozygotes in these **coverage adjusted expected genotype fractions**. By using this coverage filter and obtaining the **coverage adjusted expected genotype frequencies**, the bias that is intrinsically present in the observed data is also introduced in the **frequencies of the theoretical expected genotypes**.

This sampling procedure was conducted in two phases, with 1,000 and 1,000,000 iterations, respectively (see also section 1.1.5). Subsequently, with the obtained **coverage adjusted expected heterozygous fractions** (e.g. 1,000 or 1,000,000 data points) a null distribution is made. If for a specific locus the **observed heterozygous fraction** shows a significant discrepancy with, and thus will be smaller than, the **coverage adjusted expected heterozyous fraction**, this locus will be considered as monoallelically methylated.

1.1.5 Overview and practical implementation

All things considered, this leads to the following statistical test for locus *l*, for which two examples can be viewed in sections 1.2.1 and 1.2.2:

- For all S samples, determine the observed allele frequencies and the total coverage for locus *l*. In case these both suffice with respect to the predetermined thresholds (section 1.1.1), proceed to the next step, if not: proceed with the next locus.
- For each sample *s*, determine the coverage for locus *l* (C_{sl}) and perform the additional data correction (section 1.1.3).
- Determine the **observed fraction of heterozygotes** (section 1.1.2).
- Based on the allele frequencies, calculate the **theoretically expected genotype frequencies** (section 1.1.2).
- Subsequently, repeat *m* times (section 1.1.4):
  - For *s* going from 1 to S:
    * Select a random genotype according to chances equal to the theoretical genotype frequencies.
    * If the selected genotype is homozygous, $s = s + 1$.
    * Else, if the selected genotype is heterozygous, select from both alleles (here A and B) C_{sl} alleles, with equal chances for A and B. When C_{sl} is low, it is expected that a fraction of the heterozygotes will be observed as homozygotes due to the fact that by coincidence one of both alleles will never be picked. Thus, it is in this step that the coverage dependent bias, present in the observed fraction of heterozygotes, is also introduced in the theoretically expected fraction.
  - Determine the **coverage adjusted expected heterozygous fraction**.
- Finally, with the aid of the *m* **coverage adjusted expected heterozygous fractions**, it is possible to construct a null distribution, calculate a corresponding p-value for the observed frequency of heterozygotes and ultimately observe a possible significant discrepancy and thus the presence of monoallelic DNA-methylation (section 1.1.4).

This new statistical methodology was developed in the R statistical environment (R 2.15.2). In a first stage, the procedure was executed with 1,000 iterations (= *m*). For loci with a p-value of 0.005 or smaller the analysis was performed a second time, but now with 1,000,000 iterations. If the final p-value was smaller than the p-value corresponding with a false discovery rate (FDR) of 0.1, the monoallelic methylation on this SNP locus was called significant.

1.2 Examples

Here, two hypothetical examples are presented to provide further insight into the data-analytical framework. These examples don't consider the additional preprocessing steps of the data (filtering and correction, see sections 1.1.1 and 1.1.3) but only illustrate the strategy explained in

sections 1.1.2, and 1.1.4. The first example involves a situation where there is monoallelic DNA-methylation present at the hypothetical locus, whereas the second example illustrates the same procedure but for a situation with biallelic DNA-methylation.

1.2.1 Example 1: presence of monoallelic DNA-methylation

Suppose that for locus l there are fourteen samples available, for which the following information is picked up (see Table 1.1):

Table 1.1: Hypothetically detected nucleotides for each sample at locus l.

**Sample**	**Locus l**	**Sample**	**Locus l**
1	AAAA	8	AA
2	A	9	TTTTT
3	TTT	10	AAA
4	AA	11	AA
5	AAAAAAA	12	TT
6	A	13	AAA
7	AAA	14	TTT

Taking into account that if in a sample only one allele is detected this sample is considered homozygous (despite the possible low coverage), the **observed genotypes** are shown in Table 1.2:

Table 1.2: Observed genotypes per sample.

**Sample**	**Locus l**	**Sample**	**Locus l**
1	AA	8	AA
2	AA	9	TT
3	TT	10	AA
4	AA	11	AA
5	AA	12	TT
6	AA	13	AA
7	AA	14	TT

Apparently, the **observed heterozygous fraction** is zero in this set-up. Subsequently, based on these observed genotypes, the **allele frequencies** can be determined (p = frequency of A; q = frequency of T).

Number of nucleotides = n_{sl} = 28
Prevalence of A = $n_{sl,a}$ = 20

Prevalence of T = $n_{sl,t}$ = 8
$\rightarrow$ **p** = $\frac{20}{28}$ en **q** = $\frac{8}{28}$

With these allele frequencies and using the Hardy-Weinberg theorem, the **theoretically expected genotype frequencies** are being calculated.

AA $\rightarrow p^2 = (\frac{20}{28})^2 = \frac{400}{784}$
TT $\rightarrow q^2 = (\frac{8}{28})^2 = \frac{64}{784}$
AT $\rightarrow 2pq = 2*\frac{20}{28}*\frac{8}{28} = \frac{320}{784} \simeq \frac{2}{5}$

With the theoretically expected genotype frequencies it is easy to determine the **theoretically expected genotypes**: they are randomly chosen according to possibilities equal to the theoretically expected genotype frequencies. Table 1.3 shows the resulting theoretically expected genotypes for five of those sampling procedures.

Table 1.3: Theoretically expected genotypes resulting from five sampling procedures.

	Sampling 1	Sampling 2	Sampling 3	Sampling 4	Sampling 5
1	AT	AA	AA	AA	AT
2	AA	TT	AA	AT	AA
3	AA	AT	AT	AT	AT
4	TT	AT	TT	AT	AA
5	AA	AA	TT	TT	AA
6	AT	AT	TT	AA	AA
7	AA	AT	AA	AT	TT
8	AT	AT	AA	TT	AT
9	AA	AA	AA	AA	AT
10	AT	AA	AA	AT	AA
11	AA	AA	TT	AT	AA
12	AA	AT	AT	AT	AT
13	AA	AT	AT	AT	TT
14	TT	AT	AA	AT	AA

The order in which these genotypes are presented is of no importance and hence is at random. As a result, the **theoretically expected heterozygous fraction** for these five samplings is $\frac{4}{14}$, $\frac{8}{14}$, $\frac{3}{14}$, $\frac{9}{14}$, $\frac{5}{14}$, respectively, and are all bigger than the observed heterozygous fraction.

As explained in the previous paragraphs, now the **coverage adjusted expected genotypes** are being determined. This is achieved by introducing a similar coverage bias in the theoretically expected genotypes. For the fourteen samples, the observed coverages are 4, 1, 3, 2, 7, 1, 3, 2, 5, 3, 2, 2, 3 and 3, respectively. These coverages are for every sampling iteration randomly assigned to each one of the theoretically expected genotypes. Once more, the order in which these coverages are assigned is of no importance. For example, take the theoretically expected

genotypes of sampling 1 in Table 1.3 and assign each of the observed coverages to one of the genotypes (see columns 1 and 2 of Table 1.4). Based on these coverages, alleles are sampled from the corresponding genotype where each has an even chance of being selected. A possible outcome of this allele sampling is shown in the third column of Table 1.4. From this outcome it is now possible to determine the **coverage adjusted expected genotypes** (last column of Table 1.4).

Table 1.4: Rendering of the theoretically expected genotypes of sampling 1 from Table 1.3, the assigned coverages, the outcome of the allele sampling as well as the coverage adjusted expected genotypes.

**Theoretically expected genotypes**	**Assigned coverage**	**Possible outcome of allele sampling**	**Coverage adjusted expected genotypes**
AT	4	AATA	AT
AA	1	A	AA
AA	3	AAA	AA
TT	2	TT	TT
AA	7	AAAAAAA	AA
AT	1	A	AA
AA	3	AAA	AA
AT	2	AT	AT
AA	5	AAAAA	AA
AT	3	TTT	TT
AA	2	AA	AA
AA	2	AA	AA
AA	3	AAA	AA
TT	3	TTT	TT

From this table it is clear that in this situation the coverage adjusted expected heterozygous fraction is $\frac{2}{14}$ and hence is smaller than the theoretically expected heterozygous fraction of sampling 1 ($\frac{4}{14}$). The coverage adjusted expected fraction of heterozygotes can now be compared to the observed heterozygous fraction. In order to establish the presence of monoallelic methylation, the observed heterozygous fraction has to be smaller than the coverage adjusted expected heterozygous fraction, which is true for this example. To settle this in a more liable and statistical manner, the procedure starting from the step where the theoretically expected genotypes are determined out of the theoretically expected genotype frequencies (Table 1.3), till the final step including the determination of the coverage adjusted expected genotypes, is repeated m times. In that way it is feasible to respectively construct a null distribution of the coverage adjusted expected heterozygous fraction, calculate a corresponding p-value for the observed heterozygous fraction and finally determine the pre- or absence of monoallelic DNA-methylation. In the case that the p-value is smaller than a selected FDR, the monoallelic methylation of the locus is called significant. For these fourteen samples and using 1,000 iterations, our methodology resulted in a p-value of 0.016. The corre-

sponding null distribution of the coverage adjusted expected heterozygous fractions is shown in Figure 1.1.

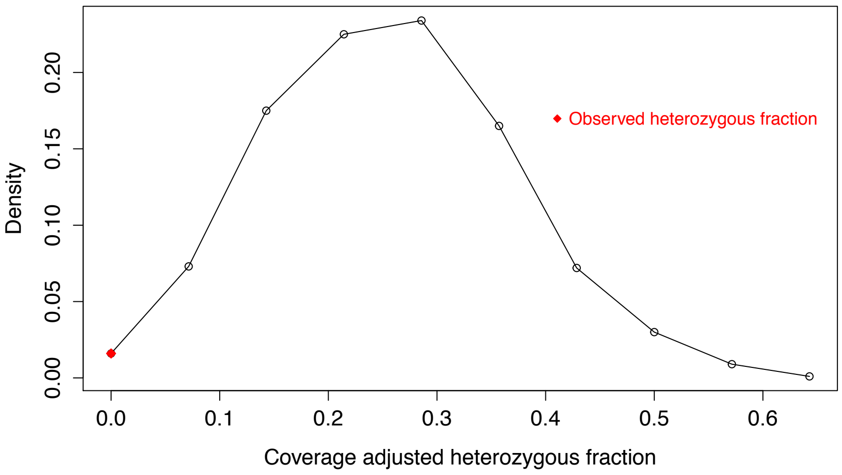

Figure 1.1: Null distribution of the coverage adjusted expected heterozygous fraction for Example 1 after 1,000 iterations.

1.2.2 Example 2: absence of monoallelic DNA-methylation

In this example the same procedure will be used as in the previous example, but now illustrating a situation in which there is no monoallelic DNA-methylation present at the hypothetical locus. Presume that the fourteen samples of locus *l* hold the following information (see Table 1.5):

Table 1.5: Hypothetically detected nucleotides for each sample at locus *l*.

Sample	Locus *l*	Sample	Locus *l*
1	AATA	8	AT
2	TTT	9	TTTAA
3	T	10	AAA
4	AA	11	AT
5	AAATTTT	12	TT
6	ATAA	13	AAT
7	TTT	14	TTT

The corresponding **observed genotypes** are illustrated in Table 1.6.

It is straightforward that in this situation the **observed heterozygous fraction** is equal to $\frac{7}{14} = \frac{1}{2}$. Based on these observed genotypes the allele frequencies can be determined (p = frequency of A; q = frequency of T).

Number of nucleotides = n_{sl} = 28
Prevalence of A = $n_{sl,a}$ = 11

Table 1.6: Observed genotypes per sample.

Sample	Locus *l*	Sample	Locus *l*
1	AT	8	AT
2	TT	9	AT
3	TT	10	AA
4	AA	11	AT
5	AT	12	TT
6	AT	13	AT
7	TT	14	TT

Prevalence of T = $n_{sl,t}$ = 17
$\rightarrow$ **p** = $\frac{11}{28}$ and **q** = $\frac{17}{28}$

In the next step, the **theoretically expected frequencies of the genotypes** are being calculated.

AA $\rightarrow p^2 = (\frac{11}{28})^2 = \frac{121}{784}$
TT $\rightarrow q^2 = (\frac{17}{28})^2 = \frac{289}{784}$
AT $\rightarrow 2pq = 2 * \frac{11}{28} * \frac{17}{28} = \frac{374}{784}$

Subsequently, the **theoretically expected genotypes** are being determined. These are randomly chosen according to chances that are equal to the theoretically expected genotype frequencies. Table 1.7 lists the theoretically expected genotypes resulting from five of those sampling procedures.

Table 1.7: Theoretically expected genotypes after five sampling procedures.

	Sampling 1	Sampling 2	Sampling 3	Sampling 4	Sampling 5
1	TT	TT	TT	AT	AA
2	AT	TT	TT	AA	AT
3	AT	AA	AT	AT	AT
4	TT	TT	AT	TT	AT
5	AT	AT	AT	AT	AA
6	AT	AT	TT	AT	TT
7	AT	TT	AT	TT	AT
8	AA	TT	AT	AT	TT
9	AT	AA	AT	AT	AT
10	AT	TT	TT	AT	AA
11	TT	AA	TT	TT	TT
12	AT	AA	AT	AT	TT
13	TT	AT	AT	AA	TT
14	AT	AT	TT	AT	TT

Thus, the theoretically expected heterozygous fractions are $\frac{9}{14}$, $\frac{4}{14}$, $\frac{8}{14}$, $\frac{9}{14}$, $\frac{5}{14}$, respectively. For this example, the observed coverages are 4, 3, 1, 2, 7, 4, 3, 2, 5, 3, 2, 2, 3 and 3, respectively,

and as in Example 1, these are now for each sampling procedure randomly assigned to one of the theoretically expected genotypes. The first two columns of Table 1.8 show a possible result of this random assignment for the theoretically expected genotypes of sampling 1 of Table 1.7. Taking these coverages into account, alleles are sampled from the theoretically expected genotypes where each allele has an even chance of being selected (see third column of Table 1.8). Finally, the last column of Table 1.8 shows the corresponding **coverage adjusted expected genotypes** of these sampled sequences.

Table 1.8: Rendering of the theoretically expected genotypes, the assigned coverages, the outcome of the sampling procedure as well as the coverage adjusted expected genotypes.

Theoretically expected genotypes	Assigned coverage	Possible outcome of allele sampling	Coverage adjusted expected genotypes
TT	4	TTTT	TT
AT	3	AAT	AT
AT	1	A	AA
TT	2	TT	TT
AT	7	AATATTA	AT
AT	4	TAAT	AT
AT	3	ATT	AT
AA	2	AA	AA
AT	5	TTATA	AT
AT	3	TAT	AT
TT	2	TT	TT
AT	2	AA	AA
TT	3	TTT	TT
AT	3	TTA	AT

In this example, the **coverage adjusted expected heterozygous fraction** is equal to $\frac{7}{14}$, again smaller than the theoretically expected heterozygous fraction of sampling 1 ($\frac{9}{14}$). The steps starting from the determination of the theoretically expected genotypes till the step where the coverage adjusted expected heterozygous fraction is determined, are repeated m times so that again a null distribution can be constructed from these m coverage adjusted expected heterozygous fractions. From this null distribution, a corresponding p-value can be obtained for the observed heterozygous fraction. For this example, the resulting null distribution of the coverage adjusted expected heterozygous fractions after 1,000 iterations is shown in Figure 1.2.

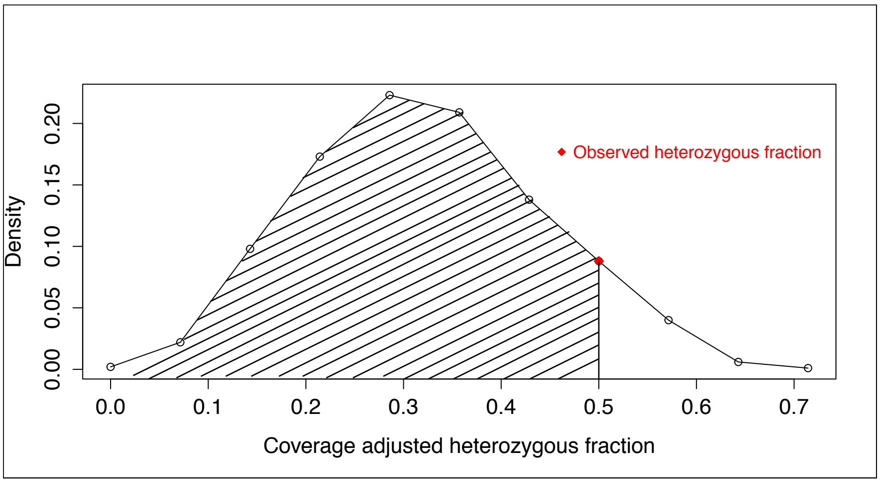

Figure 1.2: Null distribution of the coverage adjusted expected heterozygous fraction for Example 2 after 1,000 iterations.

The final p-value of the observed heterozygous fraction was 0.953. The monoallelic methylation of this locus is consequently not proven to be significantly present and is assumed to be biallelically methylated.
